# Perspectives and lessons from the Philippines’ decades-long battle with dengue

**DOI:** 10.1016/j.lanwpc.2022.100505

**Published:** 2022-06-24

**Authors:** Erika P. Ong, Arianne Justine T. Obeles, Bradley Ashley G. Ong, Ourlad Alzeus G. Tantengco

**Affiliations:** College of Medicine, University of the Philippines, Manila, Philippines

In the Philippines, dengue is probably the most well-known and feared tropical disease. The first recorded dengue epidemic in Southeast Asia occurred in Manila in 1954, and dengue has since remained endemic.^1^ In 2019, 437,563 cases were recorded in the Philippines, contributing to the highest dengue cases ever recorded globally.^2^

To address this growing problem, the Philippine government established the National Dengue Prevention and Control Program in 1993.^3^^,^^4^ The program consists of case and vector surveillance, case diagnosis and management, integrated vector management, outbreak response, health promotion and advocacy, and research. The Department of Health has been focusing on environmental control measures, reminding citizens to make the “4 o'clock habit”, which involves emptying water containers which are potential *Aedes* breeding sites everyday, and some chemical control measures (fogging during outbreaks) in its campaigns.

However, the program has struggled to meet its goals of dengue reduction.^2^^,^^3^ One significant barrier to its success is the lack of empowerment among the stakeholders in taking responsibility for dengue prevention. Another problem encountered was the challenge of eradicating local breeding sites, which are primarily water-holding containers. In areas with unreliable piped water, residents store water in such containers. Further, miscellaneous containers are commonly kept by residents as these can be used for other purposes or even sold for income. Lastly, inefficient garbage collection services may result in scattered trash that can accumulate rainwater.

In 2016, the Dengvaxia vaccine was introduced as part of the country's dengue prevention efforts.^5^ Unfortunately, nearly two years after the campaign started, Sanofi, the vaccine developer, announced that Dengvaxia might cause ‘more severe disease’ in those who have not had previous dengue infection. By this time, over 800,000 children had been indiscriminately inoculated, and public outrage ensued, with lawsuits filed against Sanofi and various government officials due to claims of children's deaths from the vaccine and government corruption. The resulting mistrust against the public health sector plunged immunization rates, precipitating a measles outbreak in 2019.

We share your hope that *Wolbachia* species, a novel form of biocontrol for arboviral diseases, can turn the tide in the decades-long battle against dengue, bypassing the barriers to vector control mentioned above. We emphasize the need for local studies regarding the safety and efficacy of this intervention in our setting. These studies can be used as evidence to include the use of *Wolbachia* in national programs and policies for dengue control. The Dengvaxia controversy reminds us not to underestimate the importance of transparency and effective health communication to inform the public regarding the safety of this intervention for people, animals and the environment to ensure the success of the program and the satisfaction of all stakeholders.

## Contributors

Erika P. Ong contributed to conceptualization, project administration, investigation, writing of the original draft and review & editing.

Bradley Ashley G. Ong contributed to conceptualization and review & editing.

Arianne Justine T. Obeles and Ourlad Alzeus G. Tantengco contributed to review & editing.

## Declaration of interests

The authors declare no conflict of interest.
